# How to conduct systematic reviews more expeditiously?

**DOI:** 10.1186/s13643-015-0147-7

**Published:** 2015-11-12

**Authors:** Alexander Tsertsvadze, Yen-Fu Chen, David Moher, Paul Sutcliffe, Noel McCarthy

**Affiliations:** Communicable Disease Control Epidemiology and Evidence, Populations, Evidence and Technologies, Division of Health Sciences, Warwick Medical School, University of Warwick, Coventry, CV4 7AL UK; Warwick Centre for Applied Health Research & Delivery (W-CAHRD), Division of Health Sciences, Warwick Medical School, University of Warwick, Coventry, CV4 7AL UK; Ottawa Methods Centre, Ottawa Hospital Research Institute, The Ottawa Hospital, General Campus, Centre for Practice Changing Research Building, 501 Smyth Road, PO BOX 201B, Ottawa, Ontario K1H 8L6 Canada; Populations, Evidence and Technologies, Division of Health Sciences, Warwick Medical School, University of Warwick, Coventry, CV4 7AL UK

**Keywords:** Systematic reviews, Rapid reviews, Risk of bias

## Abstract

Healthcare consumers, researchers, patients and policy makers increasingly use systematic reviews (SRs) to aid their decision-making process. However, the conduct of SRs can be a time-consuming and resource-intensive task. Often, clinical practice guideline developers or other decision-makers need to make informed decisions in a timely fashion (e.g. outbreaks of infection, hospital-based health technology assessments). Possible approaches to address the issue of timeliness in the production of SRs are to (a) implement process parallelisation, (b) adapt and apply innovative technologies, and/or (c) modify SR processes (e.g. study eligibility criteria, search sources, data extraction or quality assessment). Highly parallelised systematic reviewing requires substantial resources to support a team of experienced information specialists, reviewers and methodologists working alongside with clinical content experts to minimise the time for completing individual review steps while maximising the parallel progression of multiple steps. Effective coordination and management within the team and across external stakeholders are essential elements of this process. Emerging innovative technologies have a great potential for reducing workload and improving efficiency of SR production. The most promising areas of application would be to allow automation of specific SR tasks, in particular if these tasks are time consuming and resource intensive (e.g. language translation, study selection, data extraction). Modification of SR processes involves restricting, truncating and/or bypassing one or more SR steps, which may risk introducing bias to the review findings. Although the growing experiences in producing various types of rapid reviews (RR) and the accumulation of empirical studies exploring potential bias associated with specific SR tasks have contributed to the methodological development for expediting SR production, there is still a dearth of research examining the actual impact of methodological modifications and comparing the findings between RRs and SRs. This evidence would help to inform as to which SR tasks can be accelerated or truncated and to what degree, while maintaining the validity of review findings. Timely delivered SRs can be of value in informing healthcare decisions and recommendations, especially when there is practical urgency and there is no other relevant synthesised evidence.

## Background

### Role of systematic reviews

Systematic reviews (SRs) are transparent and succinct evidence synthesis summaries of empirical results of primary research studies addressing one or more questions regarding any given health problem, intervention(s) or policy decision [1, 2]. The proper conduct of SRs entails the application of predefined explicit systematic approaches to the formulation of research question(s), study eligibility criteria, search strategy (literature sources and identification of primary studies), study selection, data extraction, assessment of methodological quality (or risk of bias) of included studies, data synthesis and analysis and grading the overall quality of evidence (e.g. the GRADE approach). These approaches have been shown to minimise bias and improve the precision of review findings [3]. Over the past two decades, SRs have become an important source of high hierarchy evidence. Healthcare consumers, researchers, patients and policy makers increasingly utilise SRs to aid their decision-making process.

### Problems of timeliness and cost

The conduct of SRs can be a time-consuming, cost- and resource-intensive task, which may take on average from 6 months to several years [4–6]. This issue becomes especially problematic when clinical practice guideline developers, healthcare agencies or other decision-makers need to make informed decisions and recommendations expeditiously. For example, scientists working in the field of infectious diseases often deal with time-sensitive circumstances dictated by clinical or public health emergency. In such situations, the timeliness is of essence at both stages of evidence synthesis and development of recommendations. Recent work to support the management of the Ebola outbreak in West Africa offers an extreme example where the need for evidence to guide hand hygiene measures was achieved by accelerated SRs [7] while in other areas requiring an evidence base expert opinion was used without any dedicated form of a SR [8]. Likewise, for academically based hospitals producing hospital-based health technology assessments (HTAs) of new or emerging technologies, both timeliness and costs of producing reviews may be critical, in particular when deadlines for the conduct and delivery of HTAs are driven by the interests of manufacturers, physicians and/or patients [9].

## Main text

Three approaches taken alone or in conjunction may be considered as possible solution(s) to address the issues of timeliness in the production of SRs: (1) implement process parallelisation, (2) adapt and apply innovative technologies allowing automation and (3) modify some SR processes. Although the latter two approaches are expected to also reduce the review production costs, both may introduce some form of bias into the review. Implementing the process of parallelisation will not reduce the costs but it will not increase the risk of bias either (see Table 1).

**Table 1:** The interrelationship between the three approaches to the conduct of reviews with expected impacts on speed, costs and risk of bias

	Process parallelisation	Application of innovative technologies	Methodological modifications
Speed increased	Yes	Yes	Yes
Cost reduced	No	Yes	Yes
Risk of bias increased	No	Possibly	Possibly

### Process parallelisation

Although different steps of a SR can be carried out by two reviewers in a linear fashion, where resources permit many tasks such as study selection, data extraction and quality assessment can be divided amongst several reviewers who can perform these tasks in parallel (at least in part), thereby reducing the time needed to complete a SR. Parallelisation of SR tasks can be analogous to the process of parallel computing [10], the method used in computer technology, when any given large computing task is divided into many smaller tasks which are then computed simultaneously rather than sequentially. One example of the process parallelisation of SR tasks would be the prioritisation of screening during which potentially relevant titles/abstracts are at the top and less relevant ones at the bottom of a screening list [11]. This approach enables one team of reviewers to identify most of the relevant citations quickly, while the other team screens the remaining mostly irrelevant citations. This allows to begin and complete other SR processes such as the retrieval of full texts, data extraction and evidence synthesis more timely, i.e. in parallel with the SR steps initiated chronologically earlier (e.g. screening). Simultaneous implementation of some SR processes can be a time-saving approach whether or not the total workload is reduced. An effective parallelisation of SR processes needs to be supported by the use of a purposefully adapted computer technology [11, 12].

Highly parallelised systematic reviewing requires a team experienced in literature search, clinical epidemiology and research methodology, often working alongside advisors with clinical, statistical and economic expertise. Effective coordination and management within the review team and across the network of external experts and stakeholders are essential parts of a successful process parallelisation. The effective management of parallelisation should not affect the quality of a review produced. However, resources required to maintain such a model of reviewing can be considerable. The assessment groups undertaking health technology assessments for the National Institute for Health and Care Excellence in the UK and the Evidence-Based Centres carrying out comparative effectiveness reviews for the Agency for Healthcare Research and Quality in the US are good examples of such type of management.

### Application of innovative technologies

Current developments in innovative technologies (automated or semi-automated) applicable to the production of SRs are a promising armamentarium for reducing costs and workload in expediting the SR process [13]. Of course, all such emerging technologies need to be evaluated for their accuracy, reliability, practicality and costs. Systematic Review (SR) Toolbox, an online catalogue, provides a downloadable list of tools to support SRs (e.g. software, assessment checklists and reporting guidelines) [14].

The most efficient use and application of the machine-learning technologies would be in the areas allowing automation of specific SR processes, in particular those involving time-consuming and resource-intensive tasks such as language translation [15], study selection [11, 16–18], data extraction [19] and risk of bias assessment [20]. Some of these technologies have already been evaluated. For example, Balk and colleagues [15] tested a free web-based application (Google Translate) for the accuracy of translation from 5 languages (Chinese, Japanese, Spanish, French, and German) into English by comparing the data extracted from publications translated to English by Google Translate to data extracted from original language publications done by native speakers. The authors found that the accuracy of translation across the languages depended on an extraction item (study design and intervention yielding higher accuracy scores) and language (most of the incorrectly extracted items for articles translated from Chinese). For the task of study selection, a new semi-automated algorithmic strategy reduced the screening workload by 50 % without missing any relevant bibliographic citation [16]. Marshall et al. developed RobotReviewer, an automated machine-learning system for assessing risk of bias (RoB) for the domains included in the Cochrane RoB tool for randomised trials. The system assigns low, high or unclear RoB rating to each domain and identifies text(s) supporting these RoB judgements. The authors observed only a 10 % difference in the overall accuracy between the RoB assessments by the machine-learning system vs. published review (71.0 % vs. 78.3 %) [20]. The review by Tsafnat et al. surveyed the available tools applicable to the automation of various SR processes (e.g. the review question formulation, search strategy, study selection, data extraction, data synthesis and write-up of a review report) [12]. The authors illustrated that not all SR tasks are equally amenable to automation.

Although fully automated SRs may remain an aspiration for the near future, the current achievements in machine-learning technologies are promising steps into automation of several SR tasks which in turn will help to expedite the production and dissemination of SRs. Collaboration between SR practitioners and experts in informatics, computer sciences and linguistics will become increasingly important in harnessing the potential of automation and artificial intelligence to increase the efficiency of systematic reviewing.

### Methodological modifications

An alternative approach to synthesise evidence more expeditiously lies in modifying the SR methodology by restricting, curtailing or bypassing one or more SR steps (e.g. study eligibility criteria, search strategy, data extraction, quality assessment, data analysis), while maintaining the same degree of transparency as in traditional SRs. Although cost saving, these modifications may pose a threat to validity of the review findings. Therefore, empirical evidence informing which traditional SR steps can be accelerated or curtailed and to what degree without gravely compromising the validity of findings would be very useful.

In response to the challenge of timeliness, there has been a growing number of ‘rapid reviews’ (RRs), described as ‘literature reviews that use methods to accelerate or streamline traditional systematic review processes’ [4, 5, 21–23]. RRs are better suited for narrowly defined research questions where one or more SR steps may be reduced or omitted [4, 6, 21, 22, 24, 25].

The term ‘rapid review’ incorporates an array of products that vary greatly in their purpose, methodological rigour, comprehensiveness, resources used, transparency and the time spent for their production, ranging from 1 to 32 weeks [24, 26]. Placing these products under the same term of ‘rapid review’ may be misleading and could contribute to a lack of conceptual clarity. Some authors have provided a taxonomy and descriptions of types of RR. For example, Hartling et al. categorised RRs depending on the level of synthesis into four groups: evidence inventories, rapid responses, true RRs (those using reduced forms of SR methodology) and automated approaches [24]. Polisena and colleagues divided RRs into six groups: accelerated, condensed, focused, form of evidence synthesis, modified and tailored RRs [26]. The wide spectrum of RR products reflects differences in how the agencies (e.g. governmental, non-profit, academic research groups) and other relevant stakeholders commissioning and producing evidence synthesis reports view, define and customise the timelines, conduct, production and dissemination of RRs [6, 26]. Understandably, there is no single accepted definition of what a RR constitutes [22, 26], nor is there any formally established methodology guidance as how to conduct RRs (or any type of RR) [4].

Thus, is there sufficient evidence to reliably guide us how best to expedite SRs without compromising their validity? The majority of RR methodology overviews represent surveys that either describe or compare the methods and processes used for conducting RRs and SRs [4, 6, 21, 22, 24–26]. In contrast, the empirical evidence from studies comparing findings between RRs and SRs is insufficient [5, 24, 26]. Indeed, such evidence would be useful in informing as to which traditional SR steps can be accelerated or curtailed and to what degree, while maintaining the validity of review findings.

Over the last two decades, empirical evidence has accumulated from studies investigating different sources of bias related to specific SR tasks. For example, several authors evaluated study location strategies [27, 28], study inclusion criteria [29–33], study selection [34, 35], data extraction [36] and study quality or risk of bias assessment [37–39] as sources of bias in SRs. Notably, more recent evidence has focused on evaluating time- and resource-efficient techniques to performing specific SR tasks. For example, Sampson et al. showed that an Embase search in addition to Medline resulted in only 6 % change in the pooled effect estimate [40]. Similarly, Royle and Milne found that searches in databases additional to Cochrane Controlled Trials Register (CCTR), Medline and Embase identified only 2.4 % more studies [41]. These findings were corroborated by Cameron et al., who suggested that comprehensive literature searches may have little impact on the conclusions of a review [42]. Another study demonstrated only a slight change in the pooled effect estimates in Cochrane reviews after excluding intervention trials not found in Medline. The authors concluded that searching sources additional to Medline, particularly Embase, resulted in small incremental gains [43]. Preston and colleagues examined 302 citations included in 9 SRs of diagnostic test accuracy studies and found that 93 % of all included citations had been retrieved by searching Medline, Embase and the reference lists [44]. Some researchers agree that when timeliness is of importance, hand searching of reference lists and contacting experts can be more effective than comprehensive bibliographic database searches [45, 46].

Another area worthy of consideration is the restriction of inclusion criteria by language of publication. The inclusion of studies regardless of the language of publication would provide a more complete coverage and a greater precision of an effect estimate. However, the evidence whether or not the exclusion of non-English language study publications of conventional healthcare interventions introduces bias has been inconsistent, some authors showing meta-analyses of only English language studies yielding more conservative estimates [29], and others not demonstrating the presence of any difference [30, 32, 47]. Some authors suggested that the impact of excluding non-English language studies may depend on the topic of the review and the quality of non-English language studies [29, 31, 32]. For example, Moher and colleagues found that in SRs of conventional interventions, language restriction did not alter the review results, whereas such restrictions resulted in a substantial change in the review results of complementary and alternative medicine interventions [31]. In general, given the recent trend showing increased rates of publications in English, the language bias may not have as strong effect as before [48].

The evidence regarding the need for quality assessment of studies included in SRs is more consistent in indicating that bypassing this important step may lead to substantial bias in the review estimates [37–39, 49, 50]. A clear illustration of this phenomenon was shown in the study by Moher and colleagues, where the pooled estimate of low-quality trials, compared to high quality-trials, demonstrated 34 % greater benefit in the treatment effect [38].

Much of the above evidence has been focused on SRs of randomised trials of health interventions. While these studies have been crucial in guiding current approaches to undertaking full or reduced methodology SRs, more empirical evidence is needed as the uptake of SR methodology expands into the evaluation of other types of questions beyond clinical effectiveness (e.g. aetiology, epidemiology or genetic associations).

## Conclusion

### Future research and perspectives

In situations of clinical urgency (e.g. outbreaks and epidemics of life threatening infections), when there is no relevant systematically reviewed evidence, timely delivered SRs can be of great value in informing healthcare decisions and recommendations. SRs conducted expeditiously may also be relevant if an existing SR is in need of updating or when the available resources are limited [51, 52]. For example, Elliot and colleagues [18], proposed an alternative solution to the problem of keeping SRs and their conclusions up-to-date and accurate. The authors proposed to initiate living systematic reviews, which represent high-quality online evidence summaries, continuously updated as any new relevant evidence becomes available. Living systematic reviews are dynamic and constantly changing online-only evidence summaries that demand less intensive work over time compared to static and sporadically more resource-intensive conventional SRs. The production and publication of living systematic reviews call for modifications in the author team management style and the use of statistical methods (to minimise the rate of false positive findings due to repeated testing associated with an update).

Future empirical evidence comparing RRs to SRs and comprehensive synthesis of methodological studies exploring the magnitude of bias arising from a modification of any given SR step are needed to provide essential foundations for the development of evidence-based methodology for conducting SRs more timely. This evidence could also highlight specific SR steps or subtasks that are either of critical importance or redundant. Assessing the validity of RRs through comparison of their findings with SRs rests on the crucial assumption that current SR methodology is the gold standard which reflects the best available approach. This may be true regarding transparency and theoretical justification for instigating various standard procedures to minimise different forms of potential bias in the review process. But to what extent is current SR methodology supported by empirical evidence to guide practice, taking into account the efficiency of SR production?

Some of the new initiatives and developments in the field are likely to inform the above-mentioned gaps in knowledge. For example, Cochrane Innovations initiated the programme of Rapid Response Review, which is designed to produce expedited reviews by using ‘abbreviated’ and ‘accelerated’ SR methods, while maintaining the methodological rigour and transparency of traditional SRs. This process implies iterative interactions between the commissioners and reviewers in formulating and refining the research question and scope of the review, thereby streamlining the review process through expeditious delivery of the response to any given research question [53].

The January 2015 issue of *Systematic Reviews* has published a thematic collection of articles highlighting important developments in the RR methodology which will likely help addressing the issues related to timely production of SRs [6, 26, 54]. In February 2015, the Canadian Agency for Drugs and Technologies in Health (CADTH) hosted the Rapid Review Summit (Then, Now, and in the Future) in Vancouver, British Columbia (Canada), where about 150 participants from Canada and other countries discussed the role of RRs in informing healthcare policy and clinical decision-making. Some of the main objectives of this summit were the following: (a) to exchange information amongst stakeholders interested in RRs, (b) to promote the knowledge exchange on applications and production of RRs and (c) to elaborate and prioritise future research agenda for the development of the RR methodology [55].

In addition to making use of automation to expedite the conduct of individual SRs, collective efforts need to be made to improve the platform for the retrieval and synthesis of research information. This can be achieved through standardisation of data collection, reporting and archiving. The best examples are clinical trial registries, the EMBASE Screening Project [56] and the Systematic Review Data Repository (SRDR) [57].

We hope that ongoing and future research initiatives will generate further relevant empirical data to better inform how best to conduct and deliver SRs timely. This evidence may also indicate contexts and/or content areas where this reduced methodology could become a standard SR approach.

## Abbreviations

CADTH: Canadian Agency for Drugs and Technologies in Health
GRADE: Grading of recommendations assessment, development and evaluation
HTA: Health technology assessment
RoB: Risk of bias
RR: Rapid review
SR: Systematic review
CCTR: Cochrane Controlled Trials Register
